# The impact of hospital volume on survival in patients with locally advanced colonic cancer

**DOI:** 10.1093/bjsopen/zrac140

**Published:** 2022-11-23

**Authors:** Emma Rosander, Torbjörn Holm, Annika Sjövall, Fredrik Hjern, Caroline E Weibull, Caroline Nordenvall

**Affiliations:** Department of Molecular Medicine and Surgery, Karolinska Institutet, Stockholm, Sweden; Department of Surgery and Urology, Danderyd Hospital, Stockholm, Sweden; Department of Molecular Medicine and Surgery, Karolinska Institutet, Stockholm, Sweden; Department of Molecular Medicine and Surgery, Karolinska Institutet, Stockholm, Sweden; Department of Pelvic Cancer, Gastrointestinal (GI) Oncology and Colorectal Surgery Unit, Karolinska University Hospital, Stockholm, Sweden; Department of Surgery and Urology, Danderyd Hospital, Stockholm, Sweden; Division of Surgery, Department of Clinical Sciences, Danderyd Hospital, Stockholm, Sweden; Clinical Epidemiology Division, Department of Medicine Solna, Karolinska Institutet, Stockholm, Sweden; Department of Molecular Medicine and Surgery, Karolinska Institutet, Stockholm, Sweden; Department of Pelvic Cancer, Gastrointestinal (GI) Oncology and Colorectal Surgery Unit, Karolinska University Hospital, Stockholm, Sweden

## Abstract

**Background:**

High hospital volume has been shown associated with improved survival in patients with several cancers. The aim of this nationwide cohort study was to investigate whether hospital volume affects survival in patients with locally advanced colonic cancer.

**Methods:**

All patients with non-metastatic locally advanced colonic cancer diagnosed between 2007 and 2017 in Sweden were included. Tertiles of annual hospital volume of locally advanced colonic cancer were analysed and 5-year overall and colonic cancer-specific survival were calculated with the Kaplan–Meier method. HRs comparing all-cause and colonic cancer-specific mortality rates were estimated using Cox models adjusted for potential confounders (age, sex, year of diagnosis, co-morbidity, elective/emergency resection, and university hospital) and mediators (preoperative multidisciplinary team assessment, neoadjuvant chemotherapy, radical resection, and surgical experience).

**Results:**

A total of 5241 patients were included with a mean follow-up of 2.7–2.8 years for low- and high-volume hospitals. The number of patients older than 79 years were 569 (32.3 per cent), 495 (29.9 per cent), and 482 (26.4 per cent) for low-, medium- and high-volume hospitals respectively. The 3-year overall survival was 68 per cent, 60 per cent and 58 per cent for high-, medium- and low-volume hospitals, respectively (*P* < 0.001 from log rank test). High volume hospitals were associated with reduced all-cause and colon cancer-specific mortality after adjustments for potential confounders (HR 0.76, 95 per cent CI 0.62 to 0.93 and HR 0.73, 95 per cent CI 0.59 to 0.91, respectively). The effect remained after inclusion of potential mediators.

**Conclusions:**

High hospital volume is associated with reduced mortality in patients with locally advanced colonic cancer.

## Teaser

This was a national register-based study on the effect of hospital volume in patients with locally advanced colonic cancer that included 5241 patients. The study showed an association between high hospital volume and decreased mortality in patients with locally advanced colonic cancer.

## Introduction

Substantial attention has been paid to hospital volume as a quality marker of cancer surgery during the past decades. It may be a marker of surgical experience, and a proxy for important structural factors related to multidisciplinary management.

Several studies have presented an association between high-volume hospitals and improved short- and long-term survival in patients with pancreatic cancer and oesophageal cancer^1,2^. Surgery of these tumours is complex and postoperative intensive care is often necessary.

The importance of hospital volume in more common tumours, such as colonic cancer demanding less complex surgery, is controversial. Surgical experience and access to advanced perioperative care may not be as important in these patients. Although, there is evidence of improved overall survival in patients with breast cancer resection in high-volume hospitals, studies on colonic cancer and survival have not found the same association^3–6^; however, subgroups of patients with colonic cancer, requiring more complicated treatments, may benefit from centralization to high-volume hospitals. These subgroups include patients with metastatic disease or locally advanced tumours.

Most locally advanced tumours can be treated curatively, but they require access to neoadjuvant treatments, advanced surgery, and sometimes postoperative intensive care.

The impact of hospital volume on survival in patients with locally advanced colonic cancer has been seldom explored. The primary aim of this study was to evaluate whether hospital volume affects survival of patients with locally advanced colonic cancer. The secondary aim was to evaluate the association between overall annual hospital volume of colonic cancer surgery, regardless of tumour stage, and survival in patients with locally advanced colonic cancer.

## Methods

### National registers

The Swedish Colorectal Cancer Registry (SCRCR) collects data on all patients diagnosed with colorectal cancer and includes data on patient characteristics, and surgical and postoperative data, as well as recurrence and survival. Patients with colonic cancer have been registered since 2007 and the coverage has improved from 94 per cent in 2007 to 97 per cent in 2017^7^. The National Patient Register (NPR) includes data on inpatient care from 1964 and on outpatient doctor visits, except for primary care, from 2001^8^. The Swedish Cancer Register (SCR) was founded in 1958 and it is compulsory for all healthcare providers to report all newly detected cancer cases to the register^9^. All deaths in Sweden are registered in the Swedish Cause of Death Register (CDR)^10^.

### Patients

**Fig. 1:**
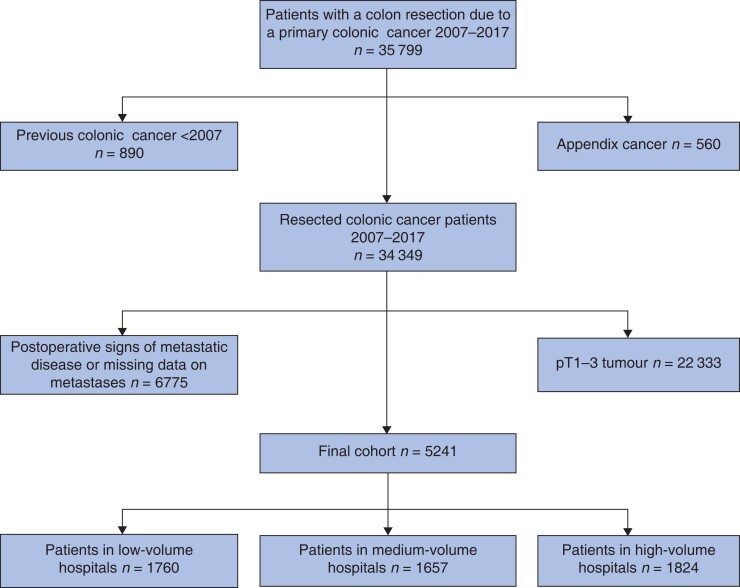
Flow chart of inclusions and exclusions in the study cohort

Data on all patients resected for colonic cancer between 2007 and 2017 in Sweden were retrieved from the SCRCR (35 799). Patients with appendiceal cancer (560), metastatic disease (6775), or pT1–3 tumours (22 333) were excluded (*Fig. 1*). Tumours registered as pT4 in the SCRCR were defined as locally advanced colonic cancer. The registers did not differ between tumours penetrating the serosa (pT4a) and tumour overgrowth to surrounding tissues (pT4b). Patients with a previous history of primary colonic cancer before the study start were excluded.

### Exposures and outcomes

The primary exposure was annual hospital volume of surgery for locally advanced colonic cancer. Data on hospitals performing this surgery were retrieved from the NPR. The hospitals were categorized into volume tertiles, yielding three mutually exclusive categories: less than 10, 11–19, and more than 19 resections per year.

As a secondary exposure, annual hospital volumes of all colonic cancer resections were used and categorized into quartiles (1–42, 43–63, 64–87, and more than 87 resections/year).

The primary outcome under investigation was 5-year all-cause mortality, and as secondary outcome, colonic cancer-specific mortality was investigated.

### Potential confounders and mediators

Age at the time of diagnosis was categorized in three groups (less than 65, 65–79, and more than 79 years old). The level of co-morbidity at diagnosis was measured using the Charlson co-morbidity index (CCI) together with ASA grade. Data from the NPR and SCR were used to calculate CCI, which was further divided into three groups: 0, 1, and more than 2 points, whereas ASA grade was divided into 1–2 and 3–5 ^11–13^. No points were given for colonic cancer. Preoperative staging of the tumour (cT) and local lymph nodes (cN) were presented according to the TNM classification. The tumour location was defined as right-sided if the tumour was in the right or transverse colon and left-sided if located from the splenic flexure to the sigmoid. Emergency resection was defined as surgery performed due to an emergent medical condition. A resection was considered radical if it was registered as microscopically radical (R0) in the SCRCR. Surgical experience was categorized into colorectal surgeon, general surgeon, and resident. Preoperative multidisciplinary team (MDT) assessment was registered as yes or no in the SCRCR. Potential confounders and mediators were illustrated in a directed acyclic graph (*Fig. S3*).

### Statistics

Time since diagnosis was used as the underlying timescale throughout, but patients started being at risk at the time of the tumour resection (delayed entry). Follow-up ended on the date of death, date of migration, or at the end of follow-up (31 December 2018), whichever came first. The Kaplan–Meier method was used to estimate survival proportions, and differences in survival between patients in different hospital volume groups were tested using the log rank test. Study population averaged survival proportions (standardized survival) were predicted from a multivariable flexible parametric survival model adjusted for sex, age at diagnosis, year of diagnosis, ASA score, CCI, emergency resection, and university hospital^14^.

Cox regression models were fitted to estimate HRs with 95 per cent confidence intervals (c.i.) contrasting all-cause and colonic cancer-specific mortality by hospital volume tertiles. Both univariable and multivariable models were fitted, where the latter were adjusted for potential confounders (sex, age at diagnosis, year of diagnosis, ASA score, CCI, emergency resection, and university hospital). Both main effects models and interaction models, including type of hospital (university hospital/non-university hospital) as an effect modifier, were fitted.

Additionally, potential mediators (preoperative MDT assessment, microscopically radical resection, neoadjuvant chemotherapy, and surgical competence) were included in the main effects models to assess the direct effect of hospital volume. All models used the clustered sandwich estimator of the standard errors, with hospital as cluster, to allow for the correlation between observations within cluster. The assumption of proportional hazards was formally tested using Schoenfeld’s residuals.

A number of sensitivity analyses were performed. First, to exclude postoperative mortality, conditional survival analyses restricted to patients who survived for more than 90 days after the tumour resection, with resection date plus 90 days as the start of follow-up, were conducted. Second, to further explore the combined effect of hospital volume and preoperative MDT assessment, a model including an interaction term between these two variables was fitted. In this analysis, hospital volume was dichotomized into high and low annual volume (1–14 and more than 14 resections per year). Third, an analysis with hospital volume categorized using annual hospital volume of all colonic cancer resections (and divided into quartiles) was performed.

Additionally, to further account for the correlation between patients treated within the same hospital, we fitted frailty models (instead of fixed-effects models) where patients treated at the same hospital shared frailty. This did not alter the results and hence will not be explored further.

Missing information on any of the variables in the model was handled using the missing indicator approach. All analyses were carried out with Stata 14/16 (StataCorp, College Station, TX, USA). The study was approved by the Regional Ethical Review Board in Stockholm (2015/1390-31, 2017/2005-32 and 2018/673-32).

## Results

### Patient and surgical characteristics

A total of 5241 patients underwent resection for locally advanced colonic cancer between 2007 and 2017 after exclusions (*Fig. 1*). Among these, 1760 patients (33.6 per cent) were treated in low-volume hospitals, 1657 patients (31.6 per cent) in medium-volume hospitals, and 1824 patients (34.8 per cent) in high-volume hospitals. Mean follow-up time was 2.7 years in low-volume hospitals and 2.8 years in medium- and high-volume hospitals. Patient and tumour characteristics are presented in *Table 1,* stratified by annual hospital volume of locally advanced colonic cancers.

**Table 1:** Preoperative patient, tumour, and treatment characteristics of 5241 patients diagnosed with locally advanced colonic cancer in Sweden between 2007 and 2017 stratified by hospital volume tertiles

	Annual pT4 colonic cancer hospital volume		
	1–10	11–19	>19
			
**Totals**	1760 (33.6)	1657 (31.6)	1824 (34.8)
**Age (years), median (range)**	75 (22–99)	74 (26–99)	73 (19–98)
**Age at diagnosis (years)**			
<65	370 (21.0)	341 (20.6)	465 (25.5)
65–79	821 (46.7)	821 (49.6)	877 (48.1)
>79	569 (32.3)	495 (29.9)	482 (26.4)
**Sex ratio (M:F)**	802 (45.6):958 (54.4)	758 (45.8):899 (54.3)	883 (48.4):941 (51.6)
**Charlson co-morbidity index**			
0	1063 (60.4)	1036 (62.5)	1134 (62.2)
1	293 (16.7)	256 (15.5)	289 (15.8)
2+	404 (23.0)	365 (22.0)	401 (22.0)
**ASA score**			
1–2	1089 (61.9)	997 (60.2)	1077 (59.1)
3–5	622 (35.3)	643 (38.8)	723 (39.6)
Missing	49 (2.8)	17 (1.0)	24 (1.3)
**Clinical tumour category (cT)**			
1–2	123 (7.0)	130 (7.9)	159 (8.7)
3	453 (25.7)	508 (30.7)	492 (27.0)
4	381 (21.7)	398 (24.0)	467 (25.6)
Missing	803 (45.6)	621 (37.5)	706 (38.7)
**Clinical nodal category (cN)**			
0	677 (38.5)	595 (35.9)	670 (36.7)
1–2	478 (27.2)	602 (36.3)	574 (31.5)
Missing	605 (34.4)	460 (27.8)	580 (31.8)
**Tumour location**			
Right colon	1072 (60.9)	1012 (61.1)	1043 (57.2)
Left colon	686 (39.0)	644 (38.9)	777 (42.6)
Missing	2 (0.1)	1 (0.1)	4 (0.2)
**Preoperative MDT assessment**			
Yes	808 (45.9)	1025 (61.9)	1201 (65.8)
No	952 (54.1)	632 (38.1)	623 (34.2)
**Neoadjuvant chemotherapy**			
Yes	39 (2.2)	48 (2.9)	75 (4.1)
No	1719 (97.7)	1607 (97.0)	1740 (95.4)
Missing	2 (0.1)	2 (0.1)	9 (0.5)
**Hospital level**			
University	184 (10.5)	314 (19.0)	737 (40.4)
Non-university	1576 (89.6)	1343 (81.1)	1087 (59.6)

Values are *n* (%) unless otherwise indicated. MDT, multidisciplinary team.

There were no significant differences in ASA score or CCI. Preoperative MDT was performed in 808 patients (45.9 per cent) in low-volume hospitals, compared with 1025 (61.9 per cent) and 1201 (65.8 per cent) patients in medium- and high-volume hospitals. The proportion of cT4 tumours were 21.7 per cent, 24.0 per cent, and 25.6 per cent respectively and the proportion treated at a university hospital ranged from 10.5 per cent in low-volume hospitals to 40.4 per cent in high-volume hospitals.

Data on surgical and postoperative details are presented in *Table 2*. Emergency surgery was performed in 546 (31.0 per cent) of the patients in low-volume hospitals, compared with 461 (27.8 per cent) and 486 (26.6 per cent) patients in medium- and high-volume hospitals. The median number of harvested lymph nodes ranged from 19 (range 0–94) in low-volume hospitals to 25 (range 1–99) in high-volume hospitals. A radical resection was performed in 1414 patients (80.3 per cent) in low-volume hospitals and 1637 patients (89.8 per cent) in high-volume hospitals. Surgery was performed by colorectal surgeons in 1494 patients (84.9 per cent) in low-volume hospitals, compared with 1509 (91.1 per cent) and 1651 (90.5 per cent) patients in medium- and high-volume hospitals.

**Table 2:** Data on surgical and postoperative details of 5241 patients diagnosed with locally advanced colonic cancer in Sweden between 2007 and 2017, stratified by hospital volume tertiles

	Annual pT4 colonic cancer hospital volume		
	1–10	11–19	>19
			
**Type of resection**			
Elective	1212 (68.9)	1193 (72.0)	1338 (73.4)
Emergency	546 (31.0)	461 (27.8)	486 (26.6)
Missing	2 (0.1)	3 (0.2)	–
**Anastomotic leakage**			
Yes	68 (3.9)	66 (4.0)	60 (3.3)
No	1692 (96.1)	1591 (96.0)	1764 (96.7)
**Stoma at tumour resection**			
Yes	351 (19.9)	332 (20.0)	482 (26.4)
No	1409 (80.1)	1325 (80.0)	1342 (73.6)
**Median number of harvested lymph nodes (range)**	19 (0–94)	20 (0–99)	25 (1–99)
**pN category**			
N0	662 (37.6)	579 (34.9)	670 (36.7)
N1–2	1090 (61.9)	1073 (64.8)	1149 (63.0)
Missing	8 (0.5)	5 (0.3)	5 (0.3)
**Radical resection**			
Radical	1414 (80.3)	1434 (86.5)	1637 (89.8)
Non-radical	342 (19.4)	218 (13.2)	179 (9.8)
Missing	4 (0.2)	5 (0.3)	8 (0.4)
**Highest surgical experience**			
Colorectal surgeon	1494 (84.9)	1509 (91.1)	1651 (90.5)
General surgeon	248 (14.1)	131 (7.9)	163 (8.9)
Resident	7 (0.4)	6 (0.4)	1 (0.1)
Missing	11 (0.6)	11 (0.7)	9 (0.5)
**Postoperative MDT assessment**			
Yes	1007 (57.2)	1298 (78.3)	1489 (81.6)
No	753 (42.8)	359 (21.7)	335 (18.4)
**Duration of hospital stay (days), median (range)**	9 (1–108)	9 (1–73)	9 (1–156)

Values are *n* (%) unless otherwise indicated. MDT, multidisciplinary team.

The number of patients admitted for adjuvant surgery was 810 (46.0 per cent) in low-volume hospitals, 799 (48.2 per cent) in medium-volume hospitals, and 964 (52.9 per cent) in high-volume hospitals (data not shown).

Resected organs are presented in *Table S1*. Adherent bowel resection was the most common multivisceral resection in all three groups (*n* = 71 (4.0 per cent), *n* = 225 (13.6 per cent), and *n* = 237 (13.0 per cent) in low-, medium- and high-volume hospitals respectively). Resection of three or more organs was performed in 50 patients (2.8 per cent) in low-volume hospitals, compared with 74 (4.5 per cent) and 102 (5.6 per cent) patients in medium- and high-volume hospitals respectively. The number of hospitals in each volume group per year is illustrated in *Fig. S1*.

### Survival analyses

The 90-day mortality rate was 4.6 per cent in high-volume hospitals, 6.5 per cent in medium-, and 6.3 per cent in low-volume hospitals (*Table S2*). The proportion of recurrent disease was 19.0 per cent, 24.8 per cent, and 26.9 per cent respectively.

**Fig. 2:**
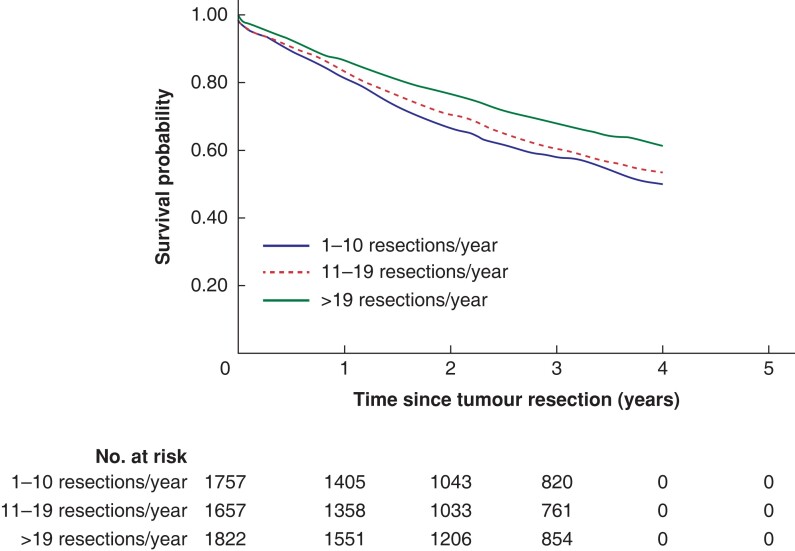
Overall survival probabilities during the first 5 years after surgery among patients resected for locally advanced colonic cancer at low-, medium-, and high-volume hospitals based on annual patient volume Data are estimated with the Kaplan–Meier method.

**Fig. 3:**
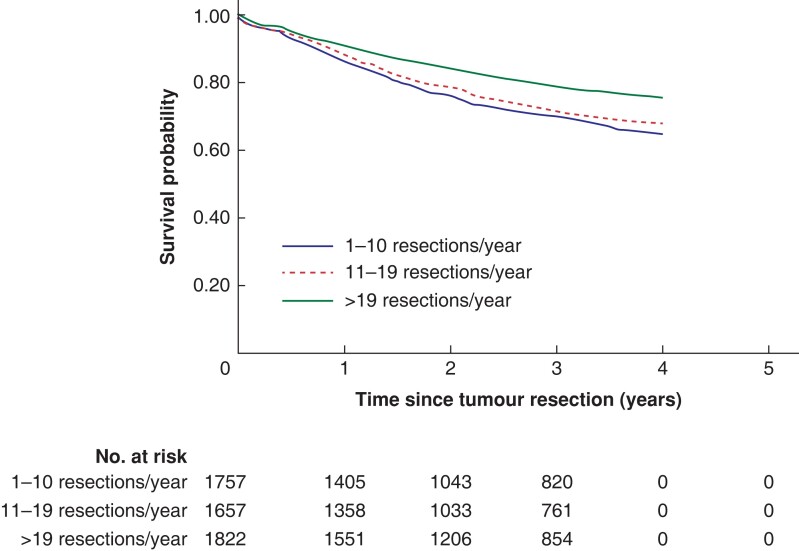
Colonic cancer-specific survival probabilities during the first 5 years after surgery among patients resected for locally advanced colonic cancer at low-, medium-, and high-volume hospitals based on annual patient volume Data are estimated with the Kaplan–Meier method.

**Fig. 4:**
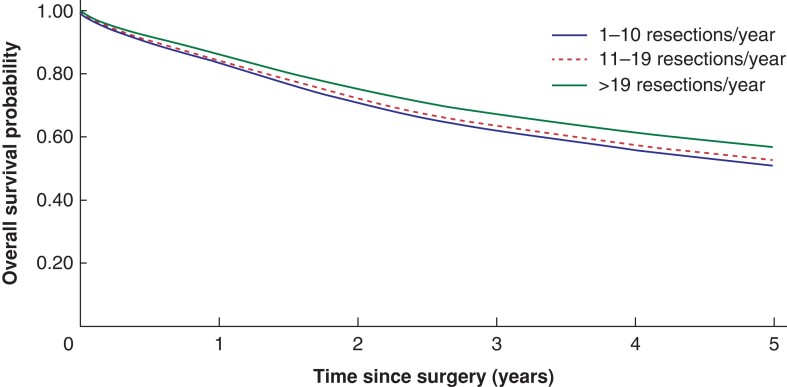
Standardized survival proportions during the first 5 years after surgery among patients resected for locally advanced colonic cancer at low-, medium-, and high-volume hospitals based on annual patient volume Data are predicted from a flexible parametric survival model adjusted for sex, age at diagnosis, year of diagnosis, university hospital, ASA score, Charlson co-morbidity index, and emergency resection.

There were significant differences in both overall and colonic cancer-specific survival by hospital volume *(Figs. 2* and *3).* Three-year overall survival was higher in high volume hospitals (68 per cent) than in medium volume and low volume hospitals (60 per cent and 58 per cent, *P* < 0.001 from log rank test). Similar differences were seen in the 3-year colon cancer specific survival (79 per cent, 71 per cent and 69 per cent for high, medium and low volume hospitals, *P* < 0.001). The study population mean survival proportions showed similar associations between high annual hospital volume and survival in locally advanced colonic cancer (*Fig. 4)*.

On the basis of multivariable models, high-volume hospitals were associated with reduced all-cause mortality after adjustments for potential confounders (HR 0.76, 95 per cent c.i. 0.62 to 0.93; *Table 3*) and additional inclusion of mediators (HR 0.81, 95 per cent c.i. 0.68 to 0.97). The effect of hospital volume differed significantly between university and non-university hospitals (*P* < 0.001 from Wald test of interaction). High- *versus* low-volume hospitals was HR 0.70 (95 per cent c.i. 0.50 to 0.98) at university hospitals, whereas for non-university hospitals the relative rate was HR 0.77 (95 per cent c.i. 0.60 to 1.00). When investigating the combination of hospital volume and preoperative MDT assessment, low volume was associated with an increased mortality without preoperative MDT assessment (HR 1.48, 95 per cent c.i. 1.09 to 1.99) compared with patients assessed before surgery in an MDT conference in high-volume hospitals *(Table S3).* In the analyses with delayed entry, the adjusted HRs remained unchanged (data not shown).

**Table 3:** HRs with 95 per cent confidence intervals comparing all-cause mortality rate between patients treated at different volume hospitals

	Univariable*	Multivariable†	Multivariable‡	Multivariable by hospital type§	
				Non-university	University
					
**Overall survival**					
Hospital pT4 volume 1–10	1	1	1	1	1
11–19	0.89 (0.81–0.98)	0.91 (0.80–1.03)	0.95 (0.84–1.07)	0.92 (0.80–1.05)	0.84 (0.59–1.20)
>19	0.71 (0.65–0.79)	0.76 (0.62–0.93)	0.81 (0.68–0.97)	0.77 (0.60–1.00)	0.70 (0.50–0.98)
**Colonic cancer-specific survival**					
Hospital pT4 volume 1–10	1	1	1	1	1
11–19	0.88 (0.78–0.99)	0.92 (0.79–1.07)	0.98 (0.84–1.14)	0.92 (0.78–1.08)	0.88 (0.56–1.40)
>19	0.65 (0.57–0.74)	0.73 (0.59–0.91)	0.81 (0.67–0.97)	0.76 (0.59–0.98)	0.66 (0.43–1.02)

Values are HR (95 per cent c.i.). Data include 5241 patients with locally advanced colonic cancer. *Estimated from a Cox proportional hazards model adjusted for the underlying timescale. †Estimated from a Cox proportional hazards model additionally adjusted for sex, age at diagnosis, year of diagnosis, university hospital, ASA score, CCI, and emergency resection. ‡Estimated from a Cox proportional hazards model adjusted as in (†) but additionally including potential mediating factors (preoperative MDT assessment, radical resection, neoadjuvant chemotherapy, and surgical experience). §Estimated from a Cox proportional hazards model adjusted as in (†) but additionally including university hospital as an effect modifier. CCI, Charlson co-morbidity index; MDT, multidisciplinary team.

The 5-year all-cause mortality by hospital volume based on the annual volume of all colonic cancers is illustrated in *Fig. S2*. There was no significant association between hospital volume and all-cause mortality after adjustments for potential confounders and mediators in the multivariable model (*Table S4)*.

## Discussion

In this nationwide study, high hospital volume was associated with an improved long-term outcome in patients with locally advanced colonic cancer and a resection rate of at least 20 resections per year was associated with decreased overall mortality. The positive effect of hospital volume remained after adjustments for potential confounders, mediators, and effect modifiers. This further emphasizes that the total effect of hospital volume cannot be explained by known mediators, such as preoperative MDT assessment, neoadjuvant chemotherapy, or radical surgery. To our knowledge this is the first study focusing on the effect of hospital volume restricted to patients with locally advanced colonic cancer.

Colorectal surgery in Sweden has gradually been centralized during the past 15 years. This has mainly affected rectal cancer, both complicated and standard cases, which today are treated in medium- and high-volume hospitals. The evidence for improved survival in patients with rectal cancer managed at high-volume hospitals is diverging^15–18^. Colonic cancer surgery has been considered as a more basic surgery that can be performed in smaller units; however, surgery of locally advanced colonic tumours should be considered as an exception. Despite the need for complex surgery and perioperative care, these patients can have the same prognosis as standard patients with colonic cancer if offered the appropriate treatment^19,20^. The spectrum of multivisceral resections is very broad. Less-complicated resections of the abdominal wall or small bowel have a postoperative recovery similar to that after standard resections. More advanced resections of the great vessels or duodenum, including the biliary tract, can be very demanding for the surgeon and are associated with severe postoperative complications. In the present study, resection of two or more organs were more common in high-volume hospitals. This can be due to selection bias if patients with more complicated locally advanced tumours are denied surgery in low-volume hospitals. On the other hand, the differences in multivisceral resections could also be explained by referral of the more complicated cases from low-volume hospitals to medium- and high-volume hospitals. High-volume hospitals were associated with improved overall survival despite more complicated multivisceral resections being conducted in this group.

There are several studies on hospital volume and colonic cancer but none focusing on patients with locally advanced colonic cancer. A US study from 2000 showed an association between hospital volume and overall survival, with the effect concentrated on colonic cancer stage II–III disease, but the external validity was low given that the cohort was restricted to Medicare-enrolled patients aged 65 years and older^21^. Another study of patients with stage I–IV colonic cancer presented a significant difference in 5-year overall survival between medium- and high-volume hospitals (52 per cent *versus* 56 per cent, *P* < 0.0118) but no association with low-volume hospitals^22^. All included hospitals participated in a quality assurance programme on a voluntary basis, which complicates the generalizability. A Cochrane analysis from 2012 presented a significant association between high-volume hospitals and improved 5-year overall survival for rectal cancer (HR 0.85, 95 per cent c.i. 0.77 to 0.93), but no such association could be found for colonic cancer^23^.

In the present study of locally advanced colonic cancer, high-volume hospitals were clearly associated with both decreased 5-year all-cause and colonic cancer-specific mortality after adjustments for potential confounders.

In the sub-analysis on hospital volume based on total annual colonic cancer volume, there was no significant association between high hospital volume and survival after adjustments for potential confounders and mediators. This indicates that volume of more complicated cases is more important for improved survival in patients with locally advanced colonic cancer than the overall volume of colonic cancer surgery.

The association between hospital volume and survival most likely depends on several factors that are important in the multimodality treatment of locally advanced tumours. Preoperative MDT assessment, correct preoperative staging, neoadjuvant chemotherapy, and surgical competence are among them. Earlier studies have shown that preoperative MDT assessment is associated with improved survival in patients with oesophageal, lung, and rectal cancer but also in locally advanced colonic cancer^24–27^. Preoperative MDT assessment was more common with increasing hospital volume. Interestingly, further adjustment for preoperative MDT assessment did not change the results. To further explore this association, a combined exposure of hospital volume and preoperative MDT assessment was computed, which showed that low hospital volume was significantly associated with increased mortality without MDT assessment. Furthermore, high hospital volume was important for long-term survival, regardless of preoperative MDT assessment and other known mediators, which further enhances the importance of centralizing patients with locally advanced disease to high-volume units.

It is likely that high-volume hospitals have better access to surgical competence specialized in colorectal surgery; however, the results remain after adjustments for surgical competence as a mediator.

University hospitals are commonly referral units for more advanced cancer disease, such as locally advanced colonic cancer. High hospital volume is not the only advantage related to an academic setting. These hospitals often have high patient volumes for different procedures, such as thoracic surgery and vascular surgery, which makes them more experienced in the perioperative management related to complex surgery. Research activities with ongoing clinical trials are also more common in university hospitals and associated with improved outcome in patients with colorectal cancer^28^; however, in the present study, the type of hospital modified the effect on overall mortality only in high-volume hospitals, and it is likely that the benefit of treatment in high-volume units cannot be explained by the type of hospital.

The major strength of this study is the large and nationwide setting, which enables the generalizability to other countries with comparable populations. The completeness of colonic cancer registration in the SCRCR was 98.5 per cent between 2008 and 2015^29^. By linkage to national patient registers the data set was completed with data on co-morbidities, previous cancer disease, and survival. Furthermore, this is the only study, to our knowledge, focusing on hospital volume in locally advanced colonic cancer surgery.

One limitation is that register information and treatment details, such as surgical quality as well as type and length of adjuvant chemotherapy, were missing. As in all studies of an observational nature there is a risk of residual confounding. For example, patients living in remote areas are more likely to be managed in low-volume hospitals than patients in urban areas. These patients are often older and have more co-morbidities. The potential differences in co-morbidity were handled by adjustments for both ASA score and CCI in the multivariable models. Lymph node harvest is an indicator of the quality of the surgery. Other possible measurements, such as the quality of the mesocolic excision, were not available in the register.

Another limitation is that the registers did not differ between pT4a and pT4b tumours. This study focused on the complex surgery of pT4b tumours, but the inclusion of pT4a tumours may have diluted the results.

In this study of patients having surgery for locally advanced colonic cancer, high hospital volume was associated with decreased mortality, and the association cannot be explained by known mediators. This knowledge should be considered in the discussion of centralization of patients with locally advanced colonic cancers.

## Supplementary Material

zrac140_Supplementary_Data

## Data Availability

The data used in the analyses include patient data, which are confidential and cannot be shared. The statistical methods used in the analyses are available upon request.
