# Supplementary material for: The impact of hospital volume on survival in patients with locally advanced colonic cancer
Source: BJS Open. 2022 Nov 23;6(6):zrac140. doi: 10.1093/bjsopen/zrac140 (PMC9683387; doi:10.1093/bjsopen/zrac140)
Supplement: zrac140_Supplementary_Data [file zrac140_supplementary_data.docx]

**Title: The impact of hospital volume on survival in patients with locally advanced colon cancer**

Rosander Emma, M.D. 1,2, Holm Torbjörn, M.D., Ph.D.1, Sjövall Annika, M.D., Ph.D.1,3, Hjern Fredrik, M.D., Ph.D. 2,5, Weibull Caroline E, Ph.D.6, Nordenvall Caroline, M.D., Ph.D.1,3

Original article

1 Department of Molecular Medicine and Surgery, Karolinska Institutet, Stockholm, Sweden

2 Department of Surgery and Urology, Danderyd University Hospital, Stockholm, Sweden

3Department of Pelvic Cancer, GI oncology and colorectal surgery unit, Karolinska University Hospital, Stockholm, Sweden

5 Division of Surgery, Department of Clinical Sciences, Danderyd Hospital, Stockholm, Sweden

6 Clinical Epidemiology Division, Department of Medicine Solna, Karolinska Institutet, Stockholm, Sweden

**Corresponding author.** Emma Rosander

**ORCID ID**; **Twitter**

**Supplementary Materials - Index**

| **Supplementary Figures and Tables** |  |
| --- | --- |
| **Figure S1.** The number of Swedish hospitals in each volume class based on locally advanced colon cancer resections/year.  **Figure S2.** Overall survival probabilities during the first five years after surgery among patients resected for locally advanced colon cancer at low, medium and high volume hospitals based on *the total annual volume of colon cancer resections*. Estimated with the Kaplan Meier method.  **Figure S3:** Directed Acyclic Graph (DAG) of potential confounders and mediators.  **Table S1.** Resected organs in 5241 patients diagnosed with locally advanced colon cancer in Sweden in 2007-2017, stratified by hospital volume tertiles.  **Table S2.** Data on recurrence during the first five years after surgery among patients resected for locally advanced colon cancer at low, medium and high volume hospitals based on annual patient volume. Including 5241 patients with locally advanced colon cancer.  **Table S3.** Hazard ratios (HRs) with 95% confidence intervals (CIs) comparing the all-cause mortality rate in combinations of hospital volume and preoperative MDT as exposure. Including 5241 patients with locally advanced colon cancer.  **Table S4.** Hazard ratios (HRs) with 95% confidence intervals (CIs) comparing the all-cause mortality rate between patients treated at different volume hospitals based on the *total colon cancer hospital volume*. Including 5241 patients with locally advanced colon cancer. | *page 3*  *page 3*  *page 4*  *page 5*  *page 5*  *page 6*  *page 6* |
|  |  |
|  |  |
|  |  |

**Supplementary Figures and Tables**

| **Figure S1.** The number of Swedish hospitals in each volume class based on locally advanced colon cancer resections/year.  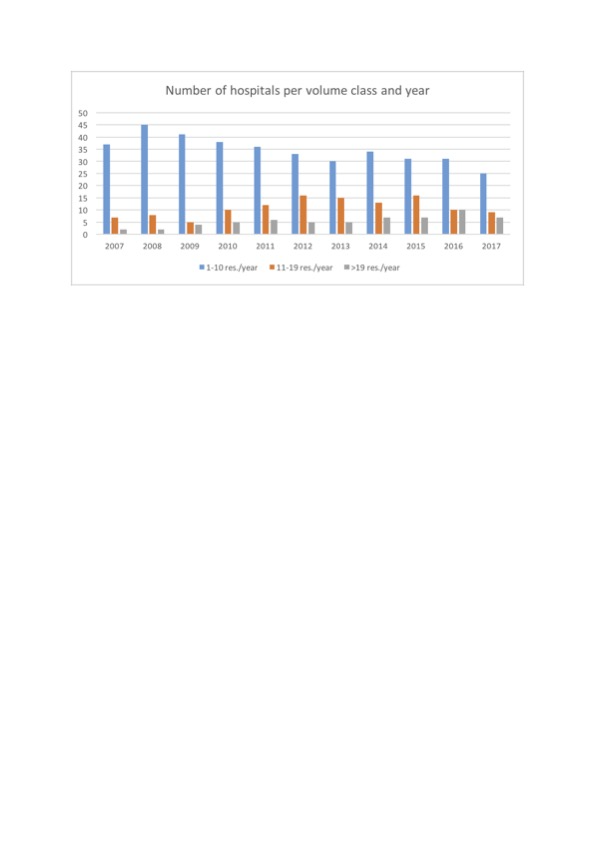  **Figure S2.** Overall survival probabilities during the first five years after surgery among patients resected for locally advanced colon cancer at low, medium and high volume hospitals based on *the total annual volume of colon cancer resections*. Estimated with the Kaplan Meier method.  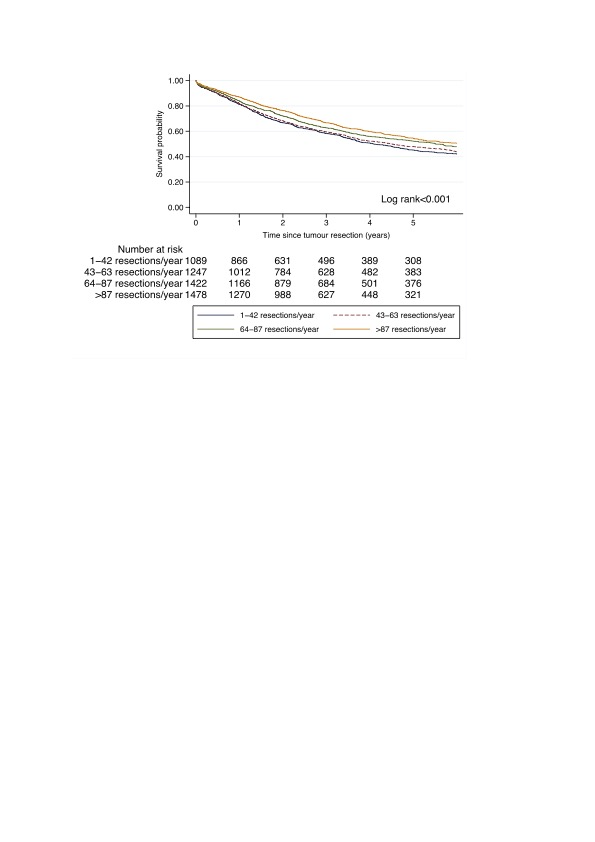  **Figure S3:** Directed Acyclic Graph (DAG) of potential confounders and mediators.  **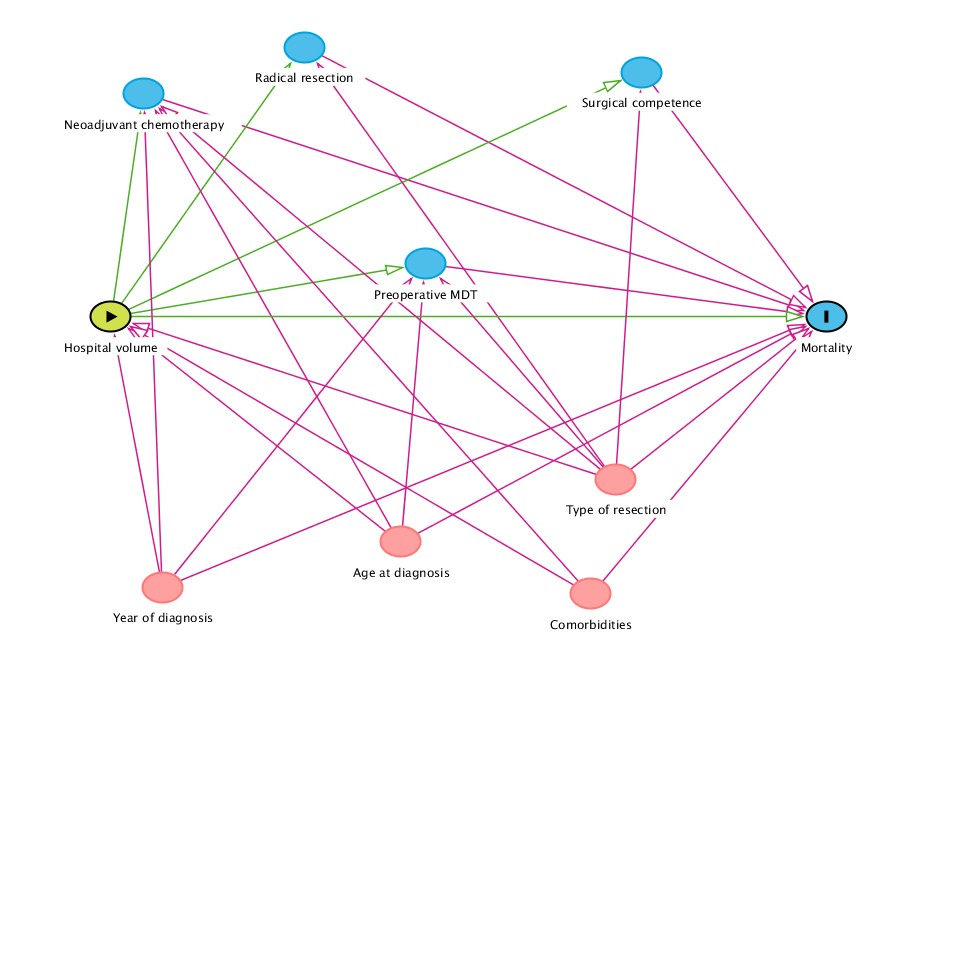**  **Table S1.** Resected organs in 5241 patients diagnosed with locally advanced colon cancer in Sweden in 2007-2017, stratified by hospital volume tertiles.   \|  \| 1-10 \| \| 11-19 \| \| >19 \| \| \| --- \| --- \| --- \| --- \| --- \| --- \| --- \| \| *n* \| *%** \| *n* \| *%** \| *n* \| *%** \| \| Organs resected** \|  \|  \|  \|  \|  \|  \| \| Abdominal wall \| 20 \| 2.6 \| 67 \| 4.0 \| 90 \| 4.9 \| \| Adherent bowel res. \| 71 \| 4.0 \| 225 \| 13.6 \| 237 \| 13.0 \| \| Part of urinary bladder \| 11 \| 0.6 \| 40 \| 2.4 \| 46 \| 2.5 \| \| Cystectomy \| 14 \| 0.8 \| 49 \| 3.0 \| 64 \| 3.5 \| \| Ovaries (uni/bilat) \| 21 \| 1.2 \| 88 \| 5.3 \| 90 \| 4.9 \| \| Uterus and/or vagina \| 7 \| 0.4 \| 36 \| 2.2 \| 46 \| 2.5 \| \| Ureter (uni/bilat) \| 6 \| 0.3 \| 15 \| 0.9 \| 16 \| 0.9 \| \| Pancreatic resection \| 7 \| 0.4 \| 12 \| 0.7 \| 20 \| 1.1 \| \| Gastric resection \| 4 \| 0.2 \| 20 \| 1.2 \| 34 \| 1.9 \| \| Splenectomy \| 8 \| 0.5 \| 12 \| 0.7 \| 29 \| 1.6 \| \| Liver resection \| 3 \| 0.2 \| 11 \| 0.7 \| 11 \| 0.6 \| \| Nephrectomy \| 1 \| 0.1 \| 9 \| 0.5 \| 9 \| 0.5 \| \| Cholecystectomy \| 5 \| 0.3 \| 17 \| 1.0 \| 14 \| 0.8 \| \| Other*** \| 1 \| 0.1 \| 5 \| 0.3 \| 7 \| 0.4 \| \| Number of organs/structures resected \|  \|  \|  \|  \|  \|  \| \| 1 \| 330 \| 18.8 \| 275 \| 16.6 \| 267 \| 14.6 \| \| 2 \| 102 \| 5.8 \| 87 \| 5.3 \| 139 \| 7.6 \| \| ≥3 \| 50 \| 2.8 \| 74 \| 4.5 \| 102 \| 5.6 \|   *Percentage of the patients in the hospital volume group  ** One patient can be registered several times if more than one resected organ  *** Coccyx, great vessels, adrenal glands  **Table S2.** Data on recurrence during the first five years after surgery among patients resected for locally advanced colon cancer at low, medium and high volume hospitals based on annual patient volume. Including 5241 patients with locally advanced colon cancer.   \|  \| 1-10 \| 11-19 \| >19 \| \| --- \| --- \| --- \| --- \| \| *n (%)* \| *n (%)* \| *n (%)* \| \| Postoperative mortality  (within 90 days) \| 111 (6.3) \| 108 (6.5) \| 84 (4.6) \| \| Total mortality \| 973 (55.3) \| 830 (50.1) \| 721 (39.5) \| \| Recurrent disease \| 474 (26.9) \| 411 (24.8) \| 346 (19.0) \| \| Local recurrence \| 177 (10.1) \| 116 (7.0) \| 77 (4.2) \| \| Distant recurrence \| 381 (21.7) \| 353 (21.3) \| 308 (16.9) \|   **Table S3.** Hazard ratios (HRs) with 95% confidence intervals (CIs) comparing the all-cause mortality rate in combinations of hospital volume and preoperative MDT as exposure. Including 5241 patients with locally advanced colon cancer.   \|  \|  \| Univariable^a^ \| Multivariable^b^ \| \| --- \| --- \| --- \| --- \| \|  \|  \|  \|  \| \|  \|  \| **HR (95% CI)** \| **HR (95% CI)** \| \| Volume>14 res./year and preop. MDT assessment \| \| 1 \| 1 \| \| Volume>14 res./year without preop. MDT assessment \| \| 1.73 (1.42-2.10) \| 1.06 (0.68—1.68) \| \| Volume1-14 res./year and preop. MDT assessment \| \| 1.35 (1.15-1.59) \| 1.25 (0.96-1.64) \| \| Volume1-14 res./year without preop. MDT assessment \| \| 2.37 (2.03-2.77) \| 1.48 (1.09-1.99) \|   ^a^ Estimated from a Cox proportional hazards model adjusted for the underlying time scale.  ^b^ Estimated from a Cox proportional hazards model additionally adjusted for sex, age at diagnosis, year of diagnosis, ASA score, CCI, and emergency resection.  **Table S4.** Hazard ratios (HRs) with 95% confidence intervals (CIs) comparing the all-cause mortality rate between patients treated at different volume hospitals based on the *total colon cancer hospital volume*. Including 5241 patients with locally advanced colon cancer.   \|  \|  \| Univariable^a^ \| Multivariable^b^ \| Multivariable^c^ \| Multivariable by hospital type^d^ \| \| \| --- \| --- \| --- \| --- \| --- \| --- \| --- \| \|  \|  \|  \|  \|  \| **Non-university** \| **University** \| \|  \|  \| **HR (95% CI)** \| **HR (95% CI)** \| **HR (95% CI)** \| **HR (95% CI)** \| **HR (95% CI)** \| \| Hospital pT4 volume \| \|  \|  \|  \|  \|  \| \| 1-42 \| \| 1 \| 1 \| 1 \| 1 \| 1 \| \| 43-63 \| \| 0.95 (0.84-1.06) \| 0.98 (0.84-1.14) \| 1.01 (0.88-1.15) \| 0.95 (0.80-1.14) \| 1.08 (0.83-1.40) \| \| 64-87 \| \| 0.84 (0.75-0.95) \| 0.89 (0.75-1.06) \| 0.95 (0.82-1.11) \| 0.88 (0.72-1.07) \| 0.97 (0.83-1.13) \| \| >87 \| \| 0.75 (0.66-0.84) \| 0.86 (0.75-0.98) \| 0.91 (0.80-1.04) \| 0.92 (0.81-1.05) \| 0.77 (0.63-0.94) \|   ^a^ Estimated from a Cox proportional hazards model adjusted for the underlying time scale.  ^b^ Estimated from a Cox proportional hazards model additionally adjusted for sex, age at diagnosis, year of diagnosis, university hospital, ASA score, CCI, and emergency resection.  ^c^ Estimated from a Cox proportional hazards model adjusted as in (b) but additionally including potential mediating factors (preoperative MDT assessment, radical resection, neoadjuvant chemotherapy).  ^d^ Estimated from a Cox proportional hazards model adjusted as in (b) but additionally including university hospital as an effect modifier. |
| --- | --- | --- | --- | --- | --- | --- | --- | --- | --- | --- | --- | --- | --- | --- | --- | --- | --- | --- | --- | --- | --- | --- | --- | --- | --- | --- | --- | --- | --- | --- | --- | --- | --- | --- | --- | --- | --- | --- | --- | --- | --- | --- | --- | --- | --- | --- | --- | --- | --- | --- | --- | --- | --- | --- | --- | --- | --- | --- | --- | --- | --- | --- | --- | --- | --- | --- | --- | --- | --- | --- | --- | --- | --- | --- | --- | --- | --- | --- | --- | --- | --- | --- | --- | --- | --- | --- | --- | --- | --- | --- | --- | --- | --- | --- | --- | --- | --- | --- | --- | --- | --- | --- | --- | --- | --- | --- | --- | --- | --- | --- | --- | --- | --- | --- | --- | --- | --- | --- | --- | --- | --- | --- | --- | --- | --- | --- | --- | --- | --- | --- | --- | --- | --- | --- | --- | --- | --- | --- | --- | --- | --- | --- | --- | --- | --- | --- | --- | --- | --- | --- | --- | --- | --- | --- | --- | --- | --- | --- | --- | --- | --- | --- | --- | --- | --- | --- | --- | --- | --- | --- | --- | --- | --- | --- | --- | --- | --- | --- | --- | --- | --- | --- | --- | --- | --- | --- | --- | --- | --- | --- | --- | --- | --- | --- | --- | --- | --- | --- | --- | --- | --- | --- | --- | --- | --- | --- | --- | --- | --- | --- | --- | --- | --- | --- | --- | --- | --- | --- | --- | --- | --- | --- | --- | --- | --- | --- | --- | --- | --- | --- | --- | --- | --- | --- | --- | --- | --- | --- | --- | --- | --- | --- | --- | --- | --- | --- | --- | --- | --- | --- | --- | --- | --- | --- | --- | --- | --- |
